# Prevalence and factors associated with high-risk thrombophilia: a single-center cross-sectional study of 3550 patients at a tertiary Thrombosis Centre in Switzerland

**DOI:** 10.1016/j.rpth.2025.102864

**Published:** 2025-04-17

**Authors:** Shabnam Najaf Zadeh, Fabienne Schmidli, Katarzyna Aleksandra Jalowiec, Tobias Tritschler, Yan Xu, Alan Haynes, Grégoire Le Gal, Anne Angelillo-Scherrer, Kristina Vrotniakaite-Bajerciene

**Affiliations:** 1 Department of Hematology and Central Hematology Laboratory, Inselspital, Bern University Hospital, University of Bern, Bern, Switzerland; 2 Department of General Internal Medicine, Inselspital, Bern University Hospital, University of Bern, Bern, Switzerland; 3 Department for BioMedical Research, University of Bern, Bern, Switzerland; 4 Department of Internal Medicine, Ottawa Hospital Research Institute, University of Ottawa, Ottawa, Ontario, Canada; 5 Clinical Trial Unit Bern, University of Bern, Bern, Switzerland

**Keywords:** arterial thrombosis, pregnancy-related morbidity, prevalence, thrombophilia, venous thromboembolism

## Abstract

**Background:**

Thrombophilia testing remains controversial, with no standardized recommendations across patient populations.

**Objectives:**

Given the clinical significance of high-risk thrombophilia (homozygous factor V Leiden or prothrombin G20210A mutations, natural anticoagulant deficiencies, and antiphospholipid antibody syndrome [APS]), we aimed to determine its prevalence and the clinical and laboratory factors associated with its diagnosis across diverse patient populations.

**Methods:**

We conducted a single-center cross-sectional study of 3550 patients tested for thrombophilia at a tertiary thrombosis clinic between 2010 and 2020. Analyses were performed in the entire cohort and by referral indication. Univariate logistic regression was used to calculate the effect measures between clinical and laboratory characteristics of referred patients and high-risk thrombophilia.

**Results:**

High-risk hereditary thrombophilia and APS were found in 155 (8%) and 67 (3%) tested patients with venous thromboembolism (VTE), in 25 (7%) and 40 (7%) tested patients with unexplained arterial thrombosis, and in 18 (17.2%) and 12 (11%) tested women with pregnancy-related morbidity, respectively. The prevalence of high-risk hereditary thrombophilia and APS was comparable in patients with unprovoked and major risk factor-provoked VTE (5.2% vs 8.2%, *P* = .1; 3.5% vs 2.8%, *P* = .9, respectively). A total of 37 (12%) of the tested asymptomatic family members had hereditary high-risk thrombophilia. Patients aged <50 years with VTE, a family history of VTE in a first-degree relative, no comorbidities, and D-dimer > 500 μg/L at the time of thrombophilia testing were more likely to have high-risk hereditary thrombophilia.

**Conclusion:**

High-risk thrombophilia was mostly prevalent in women with pregnancy-related morbidity. The prevalence of thrombophilia in patients with VTE was comparable, irrespective of VTE risk factors. Several clinical characteristics were associated with high-risk hereditary thrombophilia in patients with VTE.

## Essentials

• Thrombophilia testing remains controversial in different patient populations.
• We determined the factors associated with high-risk thrombophilia in various clinical contexts.
• The prevalence of thrombophilia was comparable between provoked and unprovoked venous thromboembolism.
• Several clinical and laboratory parameters were associated with high-risk thrombophilia.

## 1 Introduction

In recent decades, thrombophilia testing and its utility have been extensively debated. Following an initial phase of enthusiasm, interest waned, leading to underutilization [1], yet clear work-up indications remain inconclusive, and adherence to them is poor [[2], [3], [4], [5], [6]]. Not only is laboratory testing for thrombophilia technically complex and needs an advanced laboratory infrastructure, but the interpretation of the results is usually not straightforward and requires expertise [7]. The lack of uniform consensus regarding thrombophilia testing poses a great challenge for physicians in selecting patients who might benefit most from testing.

The clinical benefit of thrombophilia testing for determining anticoagulation duration is limited [8] and has been addressed in several clinical guidelines [5,9,10]. The most recent guidelines from the American Society of Hematology [11] recommend thrombophilia testing and consideration of long-term anticoagulation treatment for patients with thrombophilia and venous thromboembolism (VTE) provoked by a nonsurgical minor risk factor or exogenous estrogen use, as well as in those involving atypical sites (cerebral and splanchnic vein thromboses). While the guidance is rooted in innovative predictive outcome modeling, it faced certain limitations in methodology and occasional overgeneralizations that may affect its clinical applicability [12]. On the contrary, the recent testing guidelines from the British Society of Haematology [13] favor an intuitive and holistic approach, suggesting testing individuals aged <40 years in situations where the result could influence the treatment decision without elaborating on the exact treatment choices.

Since high-risk thrombophilia, such as hereditary high-risk thrombophilia and antiphospholipid antibody syndrome, significantly influences treatment decisions [8,14,15], identifying clinical and laboratory factors associated with these conditions across various clinical settings could increase the utility of thrombophilia testing. Given the ongoing uncertainty and debate surrounding thrombophilia testing in provoked VTE [11,13,16], data on the prevalence of high-risk thrombophilia among patients with VTE risk factors—as well as in groups like those with pregnancy-related morbidity, arterial thrombosis (ATE), or asymptomatic family members with a first-degree relative who has VTE or thrombophilia—remain limited due to selective testing focusing primarily on young patients with unprovoked VTE, as recommended by earlier guidelines [5,10,17].

The aim of this study was to investigate the prevalence and identify clinical and laboratory factors associated with high-risk thrombophilia in patients with unprovoked and provoked VTE, unexplained ATE, pregnancy-related morbidity, or asymptomatic family members with a history of VTE or thrombophilia. Using a cross-sectional study design, we analyzed patients referred to the tertiary Thrombosis Centre at the Bern University Hospital over a 10-year period.

## 2 Methods

### 2.1 Study design

This single-center cross-sectional study was conducted at the Department of Hematology of the Bern University Hospital in Switzerland – the largest thrombosis referral center in Bern. From January 2010 to October 2020, we screened consecutive patients who were referred for testing for hereditary and/or acquired thrombophilia by general practitioners or medical specialists outside of Hematology. Patients’ medical records were systematically queried in the hospital database system with the support of the hospital data management service using specific internal codes for thrombophilia work-up. Patients were included if they consented to thrombophilia testing with a documented history of objectively confirmed VTE and/or ATE in any location, a history of pregnancy-related morbidity, or a positive family history of VTE or hereditary thrombophilia. The study was approved by the Ethics Commission of the Canton of Bern (ID 2019-02102).

Standard imaging techniques were applied to diagnose venous or arterial thrombotic events [[18], [19], [20], [21], [22], [23]]. Pregnancy-related morbidities were defined as recurrent early pregnancy loss (≥3 events at less than 10 gestational weeks) and/or late pregnancy loss (≥1 at 10 or more gestational weeks), placenta insufficiency, preeclampsia [24], and HELLP syndrome (hemolysis, elevated liver enzyme levels, and low platelet count) according to diagnostic criteria of obstetricians and gynecologists [25]. VTE-provoking risk factors were categorized as minor (estrogen therapy, pregnancy, minor surgery with general anesthesia <30 minutes, and medical illness with bathroom-only privileges <3 days within 2 months prior to the diagnosis) or major (surgery with general anesthesia >30 minutes or medical illness with bathroom-only privileges >3 days, cesarean section within 3 months prior to the diagnosis, and active cancer) based on criteria from the International Society on Thrombosis and Haemostasis (ISTH) [26]. Alongside ISTH-based criteria, the presence of a central intravenous catheter [27] or May–Thurner syndrome (>70% compression of the iliac common vein) [28] was categorized as a major risk factor, whereas immobilization > 6 hours of air travel [29] and heavy smoking (>20 pack years) [30] were classified as minor risk factors. Active cancer was defined according to ISTH criteria as cancer diagnosed within the previous 6 months or for which treatment had been administered within 6 months [31].

Objective clinical data at the time of the most recent thrombotic or pregnancy-related morbidity event or at the time of the consultation for thrombophilia testing in asymptomatic family members were retrospectively collected from electronic medical records using a standardized case report form at the time of the thrombophilia work-up and entered into a computerized database in duplicate. In case of a disagreement, a third team member was included to reconcile. Data comprised demographic characteristics (age and sex at birth), a family history of VTE in first- and second-degree relatives, details of all previous thrombotic events or pregnancy-related morbidity (date and location), risk factors of the most recent VTE and ATE (heavy smoking [>20 pack years], immobilization >4 hours, infections requiring bedrest >3 days, estrogen-based medications, pregnancy and peripartum period, intravenous catheters, active cancer, obesity [body mass index > 30 kg/m^2^], trauma, surgery, cancer medication, presence of extensive varicose veins, and patent foramen ovale or other septal defects), and general comorbidities (diabetes mellitus, arterial hypertension, liver cirrhosis, kidney failure, rheumatic disease, depression, chronic inflammatory bowel disease, dyslipidemia, cardiovascular diseases, pulmonary diseases, and neurological diseases).

### 2.2 Thrombophilia and other laboratory testing

In keeping with clinical practice standards at our institution, thrombophilia testing was performed between 3 and 6 months following the index event after evaluation of the patient by a hematologist. The usual standard at the Thrombosis Centre in Bern up to 2021 was to test most of the referred patients, irrespective of their risk factors, with follow-up counseling about the results. A thrombophilia work-up was considered as “performed,” if 1 or more of the following thrombophilia parameters were tested: factor (F)V Leiden (FVL) mutation status, prothrombin gene G20210A polymorphism status, protein C (PC) and S (PS) as well as antithrombin (AT) levels, lupus anticoagulant, anticardiolipin antibodies, and anti-β2-glycoprotein I antibodies, which were usually ordered as a standard panel. Only results of accurate thrombophilia testing were considered, excluding PC and PS testing while on vitamin K antagonists or PS activity during pregnancy or while taking estrogen-containing medication. Results <70%, 59%, and 69% on at least 2 occasions were considered AT, PC, and PS deficiencies, respectively. No systematic family member testing or genetic testing was performed. The diagnosis of antiphospholipid antibody syndrome was established by persistent laboratory evidence of antiphospholipid antibodies at least 12 weeks after the first measurement and the presence of VTE, ATE, or pregnancy-related morbidity within 5 years of the most recent event [32]. Patients taking oral FXa inhibitors were instructed to interrupt the anticoagulation treatment for 48 hours before testing. No antiphospholipid antibodies were tested in asymptomatic family members with a family history of VTE or thrombophilia.

Testing for PC activity (Berichrom Protein C, Siemens; Protein C COAG, Siemens), PS antigen (Asserachrom Free Protein S, Diagnostica Stago from 2010-2015; Innovance Free Protein S Antigen, Siemens from 2015-2020), and AT activity (Coamatic LR Antithrombin, Diapharma from 2010-2013; Biophen Antithrombin LRT, Endotell from 2013-2014; and Innovance Antithrombin, Siemens from 2014-2020) was performed in the routine hemostasis laboratory (Bern University Hospital). Antiphospholipid antibodies were tested using Varelisa diagnostic kits (Phadia, Thermo Fisher Scientific) from 2010 to 2014, a fluorescence enzyme immunoassay (Phadia 250, Thermo Fisher Scientific) from 2014 to 2015, and an automated chemiluminescence assay (Bio-flash, Inova Diagnostics) from 2015 to 2020. Lupus anticoagulant was tested using a lupus-sensitive activated partial thromboplastin time assay (Hemoclot, Endotell) and confirmed by the dilute Russell’s viper venom time (CRYOcheck, Endotell). Genetic mutations were detected by the polymerase chain reaction method (FVL and prothrombin, RealFast Assay, Vienna Lab Diagnostics).

Other laboratory parameters were measured as follows: Factor VIII (FVIII:C) using one-stage coagulation assays with FVIII-deficient substrate plasma on the Attelica COAG 360 system according to the manufacturer’s (Siemens) instructions. FVIII:C > 164% was used as an upper limit of the reference range established in the Central Hematology Laboratory, University Hospital, Bern; fibrinogen concentration according to a modified Clauss method using a BCS-XP coagulometer and Multifibren U Reagent (Siemens); von Willebrand factor antigen using immunoturbidimetry on a BCS-XP device according to the manufacturer’s (Siemens) instructions; thrombin-antithrombin complex using the Human Thrombin-Antithrombin Complex ELISA Kit (TAT, Lubioscience); D-dimer with immunoturbidimetric assay (Innovance, Siemens Healthcare). The cutoffs for being positive for those assays were used according to the manufacturers.

### 2.3 Definitions of low- and high-risk thrombophilia

Low-risk hereditary thrombophilia was defined by the presence of heterozygous FVL or heterozygous prothrombin 20210G>A mutation. High-risk hereditary thrombophilia comprised homozygous FVL mutation, homozygous prothrombin 20210G>A mutation, PS, PC, and AT deficiencies or compound mutations based on their risk of first and recurrent VTE [33,34]. Antiphospholipid antibody syndrome was categorized as high-risk acquired thrombophilia.

### 2.4 Statistical analysis

Continuous and categorical variables were compared using an unpaired analysis of variance test and a chi-squared test, respectively. The associations between clinical characteristics and types of thrombophilia were evaluated using univariable logistic regression models in the entire cohort and subgroup analysis by referral indication (patients with VTE, ATE, pregnancy-related morbidity, or asymptomatic persons with a family history of VTE). Odds ratios (ORs) were calculated and reported with their 95% CIs. Only complete case analysis was performed without an attempt to replace missing values with imputation methods. A *P* value < .05 was considered statistically significant. All analyses were performed with R version 4.1.1 (R Core Team), and figures were generated using GraphPad Prism version 9.1.2 (GraphPad Software, Inc).

## 3 Results

### 3.1 Study population

Of 5064 patients screened for eligibility, we excluded 1356 patients (27%) without general consent to use their clinical data, 136 patients (3%) who were not tested for any of the thrombophilia, and 22 patients (0.4%) for the absence of objective documentation of thromboembolism, pregnancy-related morbidity, or other inclusion criteria, leaving a study sample of 3550 patients.

The main clinical characteristics of the patients according to referral indications are summarized in Table 1. The mean cohort age was 44 years (range, 18-90 years), and 2118 (60%) patients were women. The mean time between the most recent thrombotic event or pregnancy-related morbidity and thrombophilia testing was 9 months (range, 2-49 months). Most patients (2343, 66%) were referred because of VTE, mainly deep vein thrombosis and/or pulmonary embolism (1791/2343, 76%). The majority of thrombophilia testing in patients with VTE involved those provoked by a mild, transient risk factor (1245/2343, 53%). Five hundred eighty-three (583, 16.4%) patients had ATE, mainly nonembolic stroke or transitory ischemic attack (444/583, 76%). A total of 504 (14%) patients who had thrombophilia testing had no prior thromboembolic event. A minority of referrals were due to pregnancy-related morbidity (121, 3.4%). Most patients in the whole cohort had no documented comorbidities (1999, 56%) and no risk factors for VTE (1259, 35%). The most prevalent comorbidity in the cohort was arterial hypertension (578 patients, 16%), and the most common risk factor was immobilization for >4 hours (743, 21%), followed by exogenous estrogen use (706, 20%). All risk factors and comorbidities in the study cohort patients are summarized in Supplementary Table 1.

**Table 1:** Baseline characteristics of cohort patients and the referral groups.

Clinical characteristics	Whole cohort *N* = 3550 *n* (%)	Patients with VTE *n* = 2343 *n* (%)	Patients with otherwise unexplained ATE *n* = 583 *n* (%)	Females with pregnancy-related morbidity *n* = 121 *n* (%)	Asymptomatic family members *n* = 504 *n* (%)
Age^a^, y, mean (range)	44 (18-90)	46.5 (18-86)	47.2 (18-90)	34.4 (18-56)	31.6 (18-78)
Sex at birth					
Male	1432 (40)	1019 (44)	320 (55)	-	93 (18.5)
Female	2118 (60)	1324 (56)	263 (45)	121 (100)	411 (81.5)
Thrombosis risk factors^b^					
0	1259 (35)	683 (29)	150 (26)	90 (74)	333 (66)
1	1189 (33)	768 (33)	256 (44)	23 (19)	139 (27.5)
≥2	1102 (31)	892 (38)	177 (30)	8 (6.7)	32 (6.3)
Comorbidities^c^					
0	1999 (56)	1312 (56)	190 (33)	100 (83)	398 (79)
1	814 (23)	536 (23)	183 (31)	16 (13)	79 (16)
≥2	737 (21)	495 (21)	210 (36)	5 (4.2)	27 (5.4)
Family history of VTE					
First-degree relative	1106 (31)	663 (28)	108 (18.5)	30 (25)	361 (72)
Second-degree relative	523 (15)	315 (13)	40 (7)	26 (22)	143 (28)
Recurrent thrombotic event					
Yes	688 (19)	571 (24)	117 (20)	N/A	N/A
VTE context^d^					
Unprovoked	N/A	683 (29)	N/A	N/A	N/A
Minor risk factor		1245 (53)			
Major risk factor		415 (18)			
Location of thrombotic events or types of pregnancy-related morbidity					
	N/A	DVT and/or PE 1791 (76) Cerebral vein thrombosis 159 (6.8) Superficial vein thrombosis 157 (6.7) Splanchnic vein thrombosis 59 (2.5) Other^d^ 177 (7.6)	Stroke or TIA 444 (76) Thrombotic peripheral artery disease 47 (8) Thrombotic myocardial infarction 39 (7) Other^e^ 53 (9)	Fetal-related morbidity^f^ 99 (82) Placenta-related morbidity^g^ 22 (18)	N/A
Performed thrombophilia testing					
FVL	3325 (94)	2228 (95)	542 (93)	108 (88)	447 (89)
PTM	3096 (87)	2149 (92)	523 (90)	103 (85)	321 (64)
PCD	2488 (70)	1806 (77)	334 (57)	91 (75)	257 (51)
PSD	2516 (71)	1809 (77)	334 (57)	94 (78)	279 (55)
ATD	2833 (80)	2133 (91)	360 (62)	91 (75)	249 (49)
APS	2917 (82)	2240 (96)	567 (97)	110 (91)	0

Data were missing for provoking factors of VTE (0.08%), history of prior VTE at time of consultation (0.8%), and a family history of VTE in first-degree (1.3%) and second-degree (1.7%) relatives.

APS, antiphospholipid antibody syndrome; ATD, antithrombin deficiency; ATE, arterial thrombosis; DVT, deep vein thrombosis; FVL, factor V Leiden; N/A, not applicable; PCD, protein C deficiency; PE, pulmonary embolism; PSD, protein S deficiency; PTM, prothrombin gene mutation; TIA, transitory ischemic attack; VTE, venous thromboembolism.

a Age at consultation in asymptomatic patients; age at the time of the thrombotic event or pregnancy-related morbidity in other patient groups.

b Risk factors include smoking, immobilization > 4 hours, active cancer, central intravenous catheter, infection requiring bedrest with bathroom privileges only, estrogen-based treatment, pregnancy, active cancer, obesity (body mass index ≥ 30 kg/m^2^), trauma, surgery requiring systemic anesthesia at the time of the thrombotic event, or pregnancy-related morbidity or at the time of consultation in asymptomatic patients.

c Comorbidities include diabetes mellitus, arterial hypertension, the presence of any severity of liver cirrhosis, the presence of chronic kidney disease, rheumatic diseases, depression, dyslipidemia, pulmonary, neurological, and cardiovascular morbidity, and inflammatory bowel disease at the time of the thrombotic event, or pregnancy-related morbidity or at the time of consultation in asymptomatic patients.

d Other VTE locations comprise retinal vein thrombosis, kidney vein thrombosis, vena cava thrombosis, ovarian vein thrombosis, and arm vein thrombosis.

e Other arterial thrombosis locations comprise retinal artery thrombosis, kidney artery thrombosis, and spinal artery thrombosis.

f Fetal-related morbidity comprises recurrent early pregnancy loss (≥3, <10 gestational weeks) and/or late pregnancy loss (≥1, ≥10 gestational weeks).

g Placenta-related morbidity comprises (pre)eclampsia, HELLP syndrome, and placenta insufficiency.

### 3.2 Prevalence of thrombophilia

#### 3.2.1 Prevalence of high-risk thrombophilia in the entire cohort

A total of 1258 thrombophilias were detected among 1192 (34%) patients included in the overall cohort. Of these, 906 (72%) were low-risk thrombophilias, 233 (19%) were hereditary high-risk thrombophilias, and 119 (9%) were antiphospholipid antibody syndrome. The most prevalent thrombophilia was heterozygous FVL mutation (713 patients, 21.4%), followed by heterozygous prothrombin 20210G>A mutation (193 patients, 6.2%). PS deficiency was the most prevalent high-risk hereditary thrombophilia, identified in 101 (4.0%) patients (Table 2). Sixty-six patients (2%) had more than 1 thrombophilia.

**Table 2:** Prevalence of different thrombophilias among referral groups.

Type of thrombophilia	Cohort patients *N* = 3550 *n/N* (%)	Patients with VTE *n* = 2343 *n/N* (%)	Patients with ATE *n* = 583 *n/N* (%)	Pregnancy-related morbidity *n* = 121 *n/N* (%)	Asymptomatic family members *n* = 504 *n/N* (%)	*P* value
Heterozygous FV Leiden mutation	713/3325 (21.4)	480/2228 (21.5)	38/542 (7.0)	22/108 (20.4)	173/447 (38.7)	<.0001
Heterozygous 20210 G>A prothrombin mutation	193/3096 (6.2)	118/2149 (5.5)	28/523 (5.4)	9/103 (8.7)	38/321 (11.8)	.0001
Homozygous FV Leiden mutation	48/3325 (1.4)	34/2228 (1.5)	3/542 (0.6)	2/108 (1.9)	9/447 (2.0)	.22
Homozygous 20210 G>A prothrombin mutation	4/3096 (0.1)	3/2149 (0.1)	0/523	0/103	1/321 (0.3)	.64
PC < 69%	28/2488 (1.1)	18/1806 (1.0)	5/334 (1.5)	1/91 (1.0)	4/257 (1.6)	.77
PS < 59%	101/2516 (4.0)	57/1809 (3.2)	8/334 (2.4)	15/94 (15.9)	21/279 (7.5)	<.0001
AT < 70%	52/2833 (1.8)	41/2133 (1.9)	9/360 (2.5)	0/91	2/249 (0.8)	.24
Antiphospholipid antibody syndrome	119/2917 (4.1)	67/2240 (3.0)	40/567 (7.1)	12/110 (10.9)	Not tested	<.0001

Groups were compared by a chi-squared test.

AT, antithrombin; ATE, arterial thrombosis; FV Leiden; factor V Leiden; PC, protein C; PS, protein S; VTE, venous thromboembolism.

High-risk thrombophilia (hereditary, mostly represented by PS deficiency and antiphospholipid antibody syndrome) were more common in women with pregnancy-related morbidity (17% and 11%, respectively) compared with asymptomatic family members (12.2% and 0%, respectively), patients with ATE (7% and 7%, respectively), and patients with VTE (8% and 3%, respectively; *P* < .0001). All other hereditary high-risk thrombophilias were distributed equally among the groups (Table 2).

#### 3.2.2 Prevalence of high-risk thrombophilia according to clinical patients’ characteristics

Among patients with VTE (*n* = 2343), the prevalence of high-risk thrombophilias (hereditary high-risk thrombophilia and antiphospholipid antibody syndrome, accordingly) was similar in patients with VTE provoked by a transient major risk factor (8.2% and 2.8%, respectively), weakly provoked VTE (7% and 2.4%, respectively), and unprovoked VTE (5.2% and 3.5%, *P* = .6 and *P* = .4, respectively; Figure B). No difference was found in the prevalence of thrombophilia in patients with single compared with recurrent VTE (6% vs 7%, *P* = .2 in high-risk hereditary thrombophilia; 2.8% vs 3.3%, *P* = .5 in antiphospholipid antibody syndrome; Figure C). The prevalence of high-risk thrombophilia (high-risk hereditary thrombophilia and antiphospholipid antibody syndrome, accordingly) was similar in typical site VTE, including lower extremity deep vein thrombosis and/or pulmonary embolism (6.5% and 2.8%, respectively) and VTEs involving upper extremity VTE and splanchnic and cerebral vein thrombosis (6.5% and 2.8%, *P* = .9 and *P* = .2, respectively; Figure C).

**Figure:**
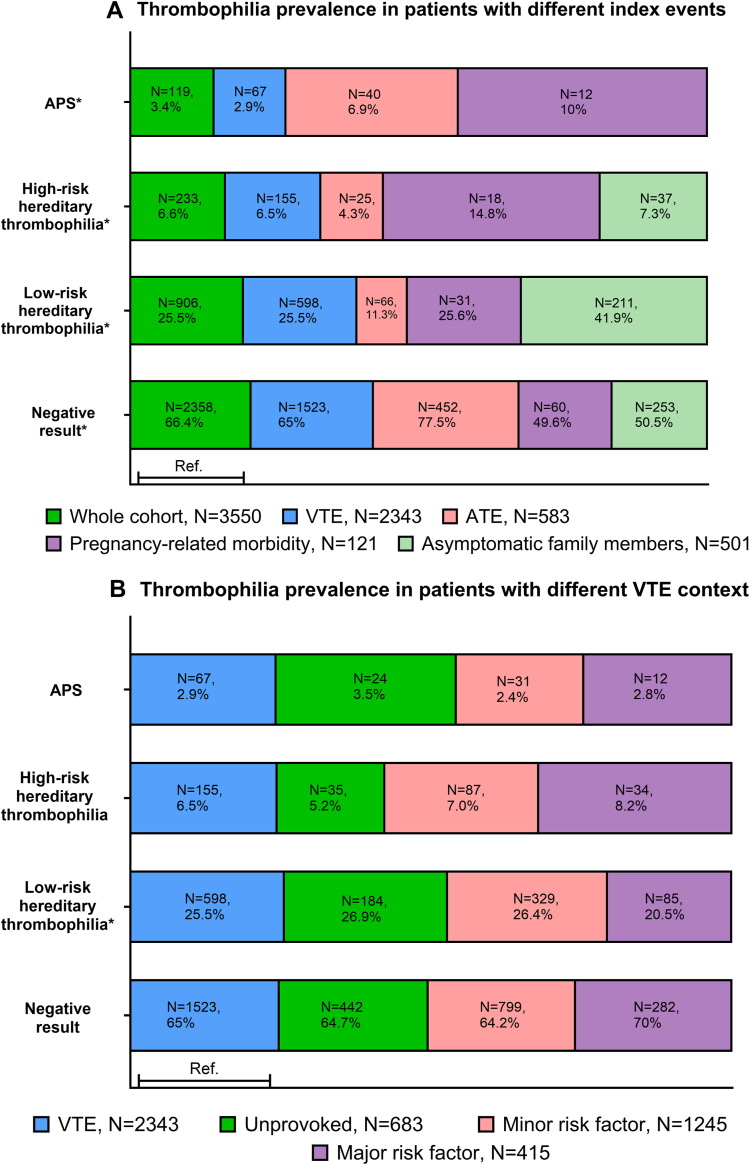

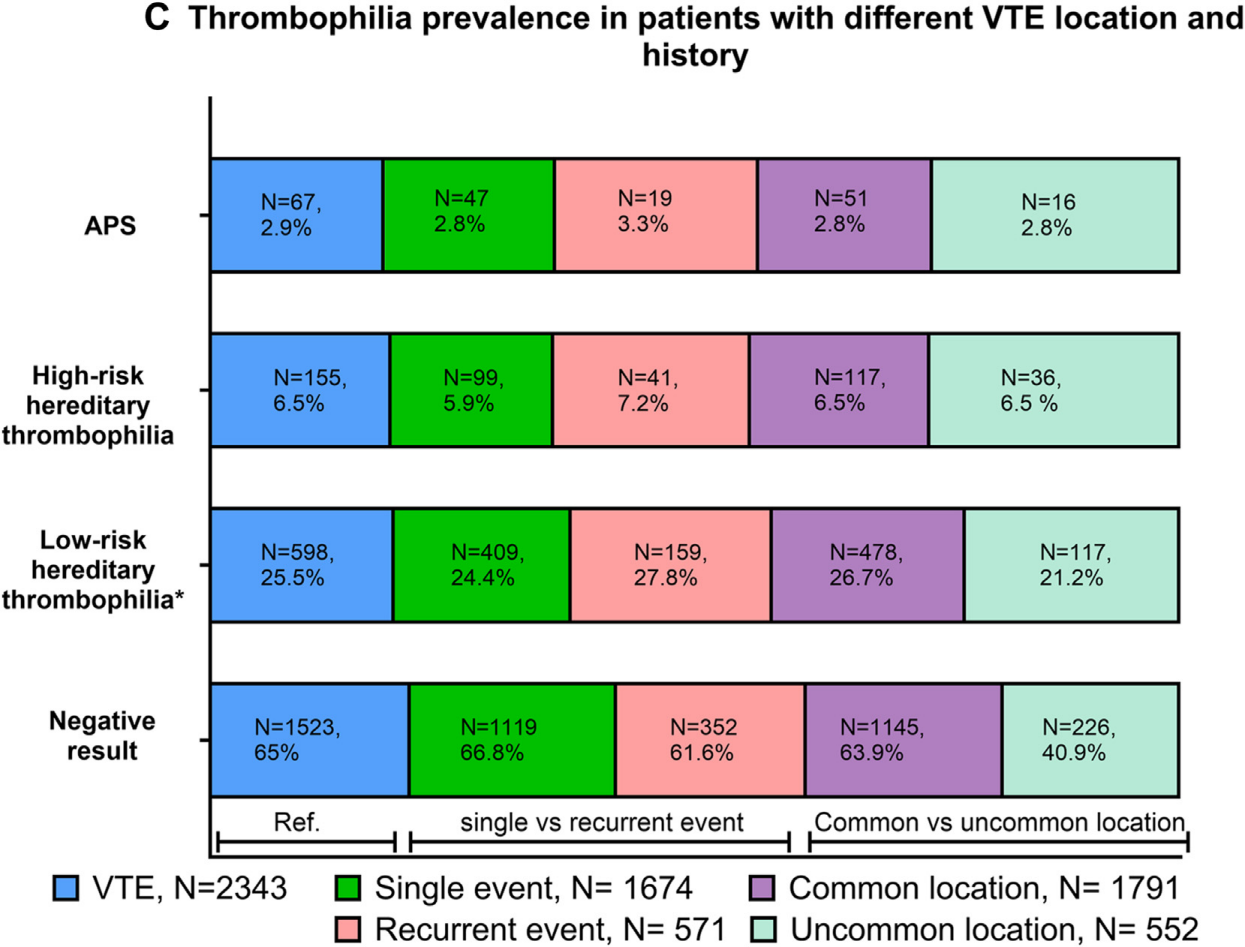
Prevalence of thrombophilia in different patient groups. (A) Prevalence of thrombophilia in the whole cohort (dark green, presented as the reference [Ref.]) and in different referral groups, including patients with venous thromboembolism (VTE; blue), otherwise unexplained arterial thrombosis (ATE; pink), pregnancy-related morbidity (purple), and asymptomatic family members with a positive family history of VTE or thrombophilia (light green). % represents the percentage of patients positive for thrombophilia within the referral group. Groups (without a Ref.) were compared using a chi-squared test. ∗Identifies significant differences in thrombophilia prevalence among patients with different referral indications within the group (*P* < .0001). Patient proportions with different thrombophilias were calculated using the whole referral group as a denominator for readability reasons. (B) Prevalence of thrombophilia in patients referred because of VTE (blue, presented as a Ref.) and according to VTE-provoking context, namely unprovoked (green), minor risk factor-provoked VTE (pink), and major risk factor-provoked VTE (purple). % represents the percentage of patients positive for thrombophilia within different VTE contexts. Groups (without a Ref.) were compared using a chi-squared test. ∗Identifies significant differences in thrombophilia prevalence (*P* < .05). The proportion of patients with no thrombophilia testing in unprovoked and minor and major risk factor-provoked VTE comprised 6%, 5%, and 5% of FVL, 10%, 7%, and 9% of prothrombin gene mutation, 10%, 8%, and 10% of antithrombin deficiency, 26%, 20%, and 25% of protein C and S deficiency, and 4%, 5%, and 4% of antiphospholipid antibody syndrome (APS), respectively. (C) The prevalence of thrombophilia in patients referred because of VTE (blue, presented as a Ref.) and according to a personal history of VTE, including a single VTE (green) and recurrent VTE (pink), and location, including a common location (purple) and uncommon location (light green). Common locations include deep vein thrombosis of the leg and pulmonary embolism. Uncommon locations include all other locations. % represents the percentage of patients positive for thrombophilia within the different VTE contexts. The proportion of patients with no thrombophilia testing in patients with a single and recurrent thrombotic event comprised 6% and 6% of FVL, 16% and 12% of prothrombin gene mutation, 21% and 19% of antithrombin deficiency, 29% and 29% of protein C and S deficiency, and 5% and 4% of APS, respectively. The proportion of patients with no thrombophilia testing in patients with a common and uncommon location event comprised 5% and 7% of FVL, 15% and 13% of prothrombin gene mutation, 21% and 19% of antithrombin deficiency, 29% and 29% of protein C and S deficiency, and 4% and 4% of APS, respectively. Groups (without a Ref.) were compared using a chi-squared test, namely recurrent vs nonrecurrent VTE and common and uncommon location VTE. ∗Identifies significant differences in thrombophilia prevalence for location (*P* < .05). Low-risk thrombophilia is defined by the presence of heterozygous factor V Leiden or heterozygous prothrombin 20210G>A mutation; high-risk hereditary thrombophilia comprises homozygous factor V Leiden, homozygous prothrombin 20210G>A mutation, protein S, protein C, and antithrombin deficiencies, or combined thrombophilia.

All types of thrombophilia were distributed equally in patients with ATE, females with pregnancy-related morbidity, and asymptomatic family members with a family history of VTE, or according to all mentioned clinical characteristics (Supplementary Table 2).

### 3.3 Predictive factors for thrombophilia

#### 3.3.1 Predictive clinical and laboratory parameters for high-risk hereditary thrombophilia

Age <50 years (OR, 1.82; 95% CI, 1.34-2.46), a positive family history of VTE in first-degree relatives (OR, 1.39; 95% CI, 1.06-1.81), and a lack of comorbidities (OR, 2.23; 95% CI, 1.50-3.32) were positively associated with a diagnosis of high-risk hereditary thrombophilia in the whole cohort (Table 3). Patients referred because of pregnancy-related morbidity were more likely to test positive for hereditary high-risk thrombophilia (OR, 2.50; 95% CI, 1.54-4.18) and less likely if referred with ATE (OR, 0.59; 95% CI, 0.38-0.92) in reference to patients with VTE (Table 3).

**Table 3:** Effect measures between clinical and laboratory characteristics of patients and positivity for low- and high-risk thrombophilia in the whole cohort and patients with venous thromboembolism.

Clinical characteristics	Low-risk hereditary thrombophilia Crude OR (95% CI)	High-risk hereditary thrombophilia Crude OR (95% CI)	Antiphospholipid antibody syndrome Crude OR (95% CI)
Cohort patients (*N* = 3550)			
Age < 50 y^a^	1.45 (1.22-1.71)	1.82 (1.34-2.46)	0.95 (0.65-1.39)
Positive family history of VTE in a first-grade relative^b^	1.78 (1.51-2.09)	1.39 (1.06-1.81)	0.64 (0.41-1.01)
Females^c^	1.16 (0.99-1.37)	1.51 (1.14-1.99)	1.16 (0.79-1.69)
Indication for consultation			
VTE	1 (ref)	1 (ref)	1 (ref)
Asymptomatic family members	2.43 (1.99-2.98)	1.11 (0.77-1.60)	-
ATE	0.42 (0.32-0.55)	0.59 (0.38-0.92)	2.50 (1.67-3.74)
Pregnancy-related morbidity	1.15 (0.76-1.76)	2.50 (1.54-4.18)	3.77 (1.98-7.19)
No. of comorbidities^d^			
0	1.98 (1.58-2.47)	2.23 (1.50-3.32)	1 (ref)
1	1.42 (1.10-1.85)	1.35 (0.86-2.17)	1.82 (1.49-2.35)
≥2	1 (ref)	1 (ref)	1.61 (1.40-1.95)
No. of thrombotic risk factors^e^			
0	1.66 (1.37-2.02)	0.94 (0.68-1.30)	1.10 (0.70-1.73)
1	1.26 (1.03-1.54)	1.10 (0.80-1.50)	1.06 (0.67-1.68)
≥2	1 (ref)	1 (ref)	1 (ref)
Patients with VTE (n = 2343)			
Age < 50 y^a^	1.25 (1.01-1.53)	2.00 (1.37-2.88)	0.03 (0.022-0.041)
Positive family history in a first-grade relative^b^	1.49 (1.20-1.86)	1.36 (1.01-1.94)	0.74 (0.38-1.34)
Females^c^	0.95 (0.78-1.17)	1.41 (1.01-2.00)	1.22 (0.72-2.09)
VTE context^f^			
Unprovoked	1 (ref)	1 (ref)	1 (ref)
Minor risk factor	1.02 (0.81-1.28)	1.31 (0.88-1.95)	0.70 (0.41-1.21)
Major risk factor	0.66 (0.48-0.91)	1.16 (0.69-1.94)	0.82 (0.40-1.65)
No. of comorbidities^d^			
0	1.34 (1.02-1.74)	1.88 (1.16-3.06)	1 (ref)
1	1.11 (0.81-1.52)	1.30 (0.73-2.31)	1.35 (0.82-2.24)
≥2	1 (ref)	1 (ref)	2.23 (1.49-3.44)
Recurrent VTE	1.36 (1.10-1.68)	1.13 (0.78-1.65)	1.21 (0.66-2.12)
D-dimer, ≥500 ug/L	1.14 (0.90-1.46)	1.78 (1.23-2.57)	0.82 (0.50-1.35)
FVIII, ≥164%	0.73 (0.56-0.94)	0.74 (0.47-1.14)	1.16 (0.71-1.90)
Fibrinogen, ≥3.75 g/L	0.97 (0.73-1.28)	0.84 (0.50-1.35)	0.71 (0.40-1.28)
Homocysteine, ≥15 μmol/L	1.01 (0.78-1.32)	0.83 (0.53-1.31)	1.15 (0.71-1.86)
VWF Ag, ≥136%	0.57 (0.21-1.53)	0.31 (0.03-3.52)	1.06 (0.46-2.45)

Adjusted ORs were calculated using univariable logistic regression, adjusting for age < 50 years and a positive family history for VTE in patients with hereditary thrombophilia. Crude ORs were calculated by univariable logistic regression in patients with antiphospholipid antibody syndrome.

Low-risk thrombophilia is defined by the presence of heterozygous factor V Leiden and heterozygous prothrombin 20210G>A mutation; high-risk thrombophilia comprises homozygous factor V Leiden mutation, homozygous prothrombin 20210G>A mutation, antithrombin < 70%, protein C < 69%, protein S < 59%, and compound thrombophilias.

ATE, arterial thrombosis; FVIII, factor VIII; OR, odds ratio; ref, reference; VTE, venous thromboembolism; VWF Ag, von Willebrand factor antigen.

a Age at consultation in asymptomatic patients, age at the time of the thrombotic event, or pregnancy-related morbidity in other patient groups.

b In reference to a negative family history of VTE in a first-grade relative.

c In reference to males.

d Comorbidities include diabetes mellitus, arterial hypertension, the presence of any severity of liver cirrhosis, the presence of chronic kidney disease, rheumatic diseases, depression, dyslipidemia, pulmonary, neurological, and cardiovascular morbidity, and inflammatory bowel disease.

e Risk factors include smoking, immobilization > 4 hours, active cancer, central intravenous catheter, infection requiring bedrest with bathroom privileges only, estrogen-based treatment, pregnancy, active cancer, obesity (body mass index ≥ 30 kg/m^2^), trauma, and surgery requiring systemic anesthesia.

f Categorization as unprovoked and minor and major provoking risk factors of VTE was based on criteria provided by the International Society on Thrombosis and Haemostasis in Kearon et al. [26].

When stratified by indications for referral, the same main clinical characteristics, such as age <50 years, a family history of VTE in first-degree relatives, and the absence of comorbidities, were associated with hereditary thrombophilia in patients with VTE (Table 3). Of note is that patients with major risk factors for provoked VTE (OR, 1.16; 95% CI, 0.69-1.94) or previous VTE events (OR, 1.13; 95% CI, 0.78-1.65) were not less likely to test positive for high-risk hereditary thrombophilia (Table 3). Main VTE risk factors were not associated with high-risk hereditary thrombophilia (Supplementary Table 3). No significant associations of clinical characteristics with high-risk hereditary thrombophilia in women with pregnancy-related morbidity, patients with otherwise unexplained ATE, and asymptomatic family members were found (Supplementary Table 4).

Regarding the main coagulation parameters at the time of consultation, patients with VTE and D-dimer elevation (>500 ug/L) at least 3 to 6 months after the index VTE event had higher odds of testing positive for high-risk hereditary thrombophilia (OR, 1.78; 95% CI, 1.23-2.57; Table 3).

#### 3.3.2 Predictive clinical and laboratory parameters for antiphospholipid antibody syndrome

Patients referred because of otherwise unexplained ATE (OR, 2.50; 95% CI, 1.67-3.74) or pregnancy-related morbidity (OR, 3.77; 95% CI, 1.98-7.19) had higher odds of testing positive for antiphospholipid antibody syndrome compared with patients with VTE. However, in contrast to hereditary thrombophilia, most clinical characteristics and laboratory parameters were not associated with antiphospholipid antibody syndrome (Table 3, Supplementary Tables 3 and 4). Only whole cohort patients with ≥1 comorbidity (OR, 1.82; 95% CI, 1.49-2.35) were more likely to test positive for antiphospholipid antibody syndrome (Table 3). This was mostly because of the presence of rheumatic disease (OR, 3.30; 95% CI, 2.00-5.44) in further detailed risk factor analysis (Supplementary Table 3).

### 3.4 Comparison of tested and untested patients included in the analysis

A total of 2504 of 3550 patients were tested for all thrombophilias (70%). The performed tests for each thrombophilia according to referral indication are presented in Table 1. Testing was not performed or missing for the presence of FVL mutation (6%), prothrombin G20210A mutation (13%), AT deficiency (20%), PC deficiency (30%), and PS deficiency (29%) in the whole cohort. Anticoagulant deficiencies were tested less frequently in patients referred with unexplained arterial events or in those referred because of a positive family history of VTE or thrombophilia (Table 1). There were no significant differences in thrombophilia testing patterns according to family history of VTE, single or recurrent thrombotic events, or among patients referred with unprovoked and minor and major risk factor-provoked VTE (≤2% difference in the proportion of patients not tested for different thrombophilias). If restricted to the analysis of patients who were tested for all thrombophilias (*n* = 2504), the prevalence of thrombophilia did not differ significantly from the initial analysis, corresponding to 497/2504 patients with FVL (20%), 129 patients with prothrombin gene mutation (5.2%), 44 patients with AT deficiency (1.7%), 24 patients with PC deficiency (0.9%), 87 patients with PS deficiency (3.5%), and 84 patients with antiphospholipid antibody syndrome (3.3%).

## 4 Discussion

In this large, single-center, cross-sectional cohort study of 3550 patients, the prevalence of high-risk hereditary thrombophilia was 8.4%, and antiphospholipid antibody syndrome was found in 4.1% of cohort patients. High-risk thrombophilia was more prevalent in women with pregnancy-related morbidity (17% and 11%, respectively) than other patient groups referred for thrombophilia testing, namely patients with VTE, otherwise unexplained ATE, or asymptomatic family members with a family history of VTE or thrombophilia. Age <50 years, a family history of VTE in first-degree relatives, absence of comorbidities, and D-dimer elevation >500 μg/L at the time of thrombophilia testing were associated with high-risk hereditary thrombophilia in patients with VTE. On the contrary, the presence of 1 or more comorbidities was associated with the diagnosis of antiphospholipid antibody syndrome. Moreover, the prevalence of high-risk thrombophilia, both hereditary and acquired, was comparable in patients with unprovoked and weak or major risk factor-provoked VTE.

High-risk thrombophilia, including both acquired and hereditary conditions, was most frequently observed in females with pregnancy-related complications (28%; 17% with hereditary thrombophilia and 11% with antiphospholipid antibody syndrome). Significant variations are found among population-based studies and clinical trials reporting the prevalence of thrombophilia in pregnancy-related morbidity, ranging from 1% to 15% [[35], [36], [37], [38]] for high-risk hereditary thrombophilia and from 5% to 38.3% for antiphospholipid antibody syndrome [[36], [37], [38], [39], [40]]. Pregnancy-related morbidity in our cohort comprised mostly recurrent early and late pregnancy losses (82%), and only 18% of the females had a documented placenta-related pathology. Therefore, we report a slightly higher prevalence of hereditary high-risk thrombophilia, mostly driven by a high frequency of PS deficiency, in women with fetal-related morbidity than reported in recent meta-analyses [41,42]. A comparable prevalence of high-risk hereditary thrombophilia in our study has been reported in populations of non-European ancestry [43] and in women with late placenta-mediated pregnancy loss [44,45]. Since preanalytical errors were excluded from the study and all PS levels were measured outside pregnancy, our study supports data on the possible role of PS deficiency besides the already established antiphospholipid antibody syndrome in fetal-related pregnancy morbidity.

The study was comprised mostly of patients with VTE (*n* = 2343, 66%) who were tested regardless of the presence of VTE risk factors. Eleven percent (11%) of the patients with VTE in our study were found to have high-risk thrombophilia, namely 8% hereditary high-risk thrombophilia and 3% antiphospholipid antibody syndrome. The prevalence of hereditary high-risk thrombophilia and antiphospholipid antibody syndrome was comparable in unprovoked, mildly provoked, and major risk factor-provoked VTE in our study. Although thrombophilia testing in both provoked and unprovoked VTE was discouraged by many guidelines given the uncertainty about the role of thrombophilia in therapy decision-making [5,9,10,17], the modern long-term VTE treatment strategy is shifting toward an individualized thrombotic risk assessment rather than mechanistic VTE dichotomization (provoked vs unprovoked) to guide the treatment choices [46]. This is also supported by the recent RIETE Registry findings showing significantly higher recurrent VTE rates in thrombophilia-positive patients with provoked VTE, including major risk factors, after discontinuing anticoagulation than that in thrombophilia-negative patients [16] and post hoc analysis from the EINSTEIN-CHOICE trials showing comparable recurrence rates of weakly provoked VTE, including patients with thrombophilia, to unprovoked VTE in patients without anticoagulation treatment [47]. Moreover, the recent recurrent VTE and bleeding case-fatality rate, as well as the quality of life analysis of patients with unprovoked VTE on long-term anticoagulation treatment, shows a negative benefit-harm trade-off of the prolonged treatment strategy [48]. Therefore, given the comparable prevalence of high-risk thrombophilia regardless of the presence of VTE risk factors, knowing the thrombophilia status could facilitate a better and more individual risk assessment process in both provoked and unprovoked VTE. The usefulness of less selective testing can be better defined in the future, however, the findings of this study are in line, suggesting the possible benefit of thrombophilia testing in patients with all types of VTE.

To increase the yield of thrombophilia testing, we attempted to identify factors associated with high-risk thrombophilia. The most meaningful analysis was related to patients with VTE and hereditary high-risk thrombophilia because of the sufficient sample size for such calculations. Heterogeneous and conflicting data in the literature exist reporting these effect measures. First, although previous studies support that individuals aged <50 years with VTE are more likely to have any type of thrombophilia [[49], [50], [51], [52]], a comparable prevalence in the elderly population has been recently reported [53,54]. Second, some studies demonstrate an association between unprovoked VTE and a positive family history of VTE in first-degree relatives with any type of thrombophilia [50,52,55], whereas others found no such association [49,51,54,56,57]. Our data support that not only age <50 years and a positive family history of VTE in first-degree relatives, but also the absence of any comorbidities at the time of VTE, were associated with high-risk hereditary thrombophilia. In our study, we also found a positive association between D-dimer elevation at the time of thrombophilia testing and the presence of high-risk hereditary thrombophilia (OR, 1.70; 95% CI, 1.24-2.32). Although, this finding is not sufficient to draw firm conclusions to justify thrombophilia testing in all patients with elevated D-dimer, it may imply the possible role of thrombophilia testing in patients before applying personalized risk prediction models, such as the HERDOO2 decision rule [58], which was derived and validated in patients without high-risk thrombophilia. Moreover, Palareti et al. [59] reported a very high risk of VTE recurrence in patients with thrombophilia and D-dimer elevation compared with carriers with a normal D-dimer, with a corresponding hazard ratio of 8.43 (95% CI, 2.72-17.43) after discontinuation of anticoagulation treatment. Thus, it may be useful to exclude this type of thrombophilia before discontinuing anticoagulation in patients with isolated D-dimer elevation.

The study has some limitations. First, its retrospective design precludes the comprehensiveness of a full thrombophilia work-up. Around 30% of patients were not tested for PC and PS, and 20% for AT. Moreover, a very small proportion of patients with PS type II deficiency and AT defects, such as AT Dublin, AT Wibble, AT Rouen VI, AT Cambridge II, AT Denver, and AT Stockholm, might have been missed because no systematic measurement of PS activity, anti-FIIa based assays, or systematic genetic testing was performed. Therefore, the prevalence of thrombophilia may be underestimated. Second, retrospective data collection from medical records may be influenced by information and recall bias; however, cross-validation of the data by 2 individuals likely limited missing values and random misclassification. Third, the study has referral bias. The cohort reflects already selected patients referred for testing by primary care physicians to a tertiary center and included a relatively small number of patients with major risk factors for VTE (415 [18%] of patients with VTE). However, 97% of referred patients were tested for any thrombophilia, and 70% had a full work-up of all types of thrombophilia, leading to a comprehensive testing pattern, which is rare in patients with provoked VTE. Moreover, a consistent definition for hereditary high-risk thrombophilia and antiphospholipid antibody syndrome was applied during the study period because of the prospective evaluation of the presence of thrombophilia according to the collected laboratory results.

In conclusion, the prevalence of high-risk thrombophilia, mostly represented in PS deficiency and antiphospholipid antibody syndrome, was highest in women with pregnancy-related morbidity. Moreover, the prevalence of high-risk thrombophilia was similar in patients with VTE regardless of VTE risk factors. Additionally, high-risk hereditary thrombophilias were associated with age <50 years, a family history of VTE, absence of comorbidities, and elevated D-dimer levels at the time of testing in patients referred for VTE. These findings suggest that thrombophilia testing could be beneficial for this patient group, also in the presence of VTE risk factors. While current guidelines may not directly suggest prolonged treatment for these patients, testing could support shared decision-making, allowing for a more comprehensive assessment of individual thrombotic risk.
