# Supplementary material for: Prevalence and factors associated with high-risk thrombophilia: a single-center cross-sectional study of 3550 patients at a tertiary Thrombosis Centre in Switzerland
Source: Res Pract Thromb Haemost. 2025 Apr 17;9(3):102864. doi: 10.1016/j.rpth.2025.102864 (PMC12138441; doi:10.1016/j.rpth.2025.102864)
Supplement: Supplementary Tables 1-4 [file mmc1.docx]

**Supplementary Table 1.** Thrombotic risk factor and co-morbidities of cohort patients

| **Clinical characteristics**  **N (%)** | **Whole cohort**      **N=3550** | **Patients with VTE**      **N=2343** | **Patients with otherwise unexplained ATE**    **N=583** | **Females with pregnancy-related morbidity**  **N=121** | **Asymptomatic family members**    **N=504** |
| --- | --- | --- | --- | --- | --- |
| Risk factors | | | | | |
| Immobilization > 4 hours | 743 (21) | 719 (30.7) | 20 (3.4) | 0 | 4 (0.8) |
| Estrogen-based medication | 706 (20) | 581 (24.8) | 66 (11.3) | 2 (1.7) | 57 (11.3) |
| Obesity (BMI > 30 kg/m2) | 576 (16) | 457 (19.5) | 82 (14.1) | 10 (8.3) | 27 (5.4) |
| Smoking | 604 (17) | 337 (14.4) | 206 (35.3) | 8 (6.7) | 53 (10.5) |
| Surgery requiring systemic anesthesia | 223 (6.3) | 213 (9.1) | 7 (1.2) | 0 | 3 (0.6) |
| PFO or another cardiac septal defect | 218 (6.1) | 26 (1.1) | 191 (32.8) | 0 | 1 (0.2) |
| Extended varicose veins | 170 (4.8) | 133 (5.7) | 18 (3.1) | 6 (5.0) | 13 (2.6) |
| Pregnancy | 153 (4.3) | 84 (3.6) | 9 (1.5) | 20 (16.7) | 40 (7.9) |
| Trauma | 135 (3.8) | 130 (5.5) | 4 (0.7) | 0 | 1 (0.2) |
| Active cancer | 110 (3.1) | 89 (3.8) | 16 (2.7) | 0 | 5 (1.0) |
| Infection requiring immobilization with bathroom privileges only | 80 (2.3) | 69 (2.9) | 10 (1.7) | 1(0.8) | 0 |
| Any cancer-related medication | 45 (1.3) | 36 (1.5) | 7 (1.2) | 0 | 2 (0.4) |
| Post-partum period | 24 (0.68) | 24 (1.0) | 0 | 0 | 0 |
| Central intravenous catheter | 19 (0.54) | 18 (0.8) | 1 (0.2) | 0 | 0 |
| Co-morbidities | | | | | |
| Arterial hypertension | 578 (16) | 395 (16.9) | 157 (26.9) | 4 (3.3) | 22 (4.4) |
| Dyslipidemia | 405 (11) | 190 (8.1) | 196 (33.6) | 4 (3.3) | 15 (3.0) |
| Pulmonary disease ^a^ | 277 (7.8) | 199 (8.5) | 55 (9.4) | 3 (2.5) | 20 (4.0) |
| Rheumatic disease | 218 (6.1) | 163 (7.0) | 34 (5.8) | 5 (4.2) | 16 (3.2) |
| Depression | 189 (5.3) | 125 (5.3) | 49 (8.4) | 0 | 15 (3.0) |
| Cardiovascular disease ^b^ | 181 (5.1) | 117 (5.0) | 58 (9.9) | 1 (0.8) | 5 (1.0) |
| Diabetes Mellitus | 138 (3.9) | 99 (4.2) | 36 (6.2) | 0 | 3 (0.6) |
| Neurological disorders ^c^ | 114 (3.2) | 82 (3.5) | 21 (3.6) | 0 | 11 (2.2) |
| Chronic kidney disease | 108 (3.0) | 86 (3.7) | 18 (3.1) | 3 (2.5) | 1 (0.2) |
| Chronic inflammatory bowel disease | 55 (1.5) | 42 (1.8) | 9 (1.5) | 0 | 4 (0.8) |
| Liver cirrhosis | 27 (0.76) | 23 (1.0) | 3 (0.5) | 0 | 1 (0.2) |

Abbreviations: ATE, arterial thrombosis; PFO, patent foramen ovale; VTE, venous thromboembolism

1. Asthma, interstitial lung disease, obstructive lung disease, obstructive sleep apnea
2. Cardiomyopathy, aortopathy
3. Epilepsy, parkinson disease, multiple sclerosis

**Supplementary Table 2.** Prevalence of low-risk and high-risk thrombophilia among patients with otherwise unexplained arterial thrombosis, asymptomatic family members with family history for venous thromboembolism or thrombophilia, and females with pregnancy-related morbidity

| Patients with otherwise unexplained ATE (N=583) | | | | |
| --- | --- | --- | --- | --- |
| Clinical Characteristics  N (%) | Low-Risk Hereditary Thrombophilia  n=66 | High-Risk Hereditary Thrombophilia  n=25 | Antiphospholipid Antibody Syndrome  n=40 | No Thrombophilia  n=452 |
| Recurrent thrombotic event  Yes | 11 (17.5) | 5 (20) | 9 (22.5) | 83 (18.2) |
| Number of thrombotic risk factors ^a^  0  1  >=2 | 18 (27.3)  29 (43.9)  19 (28.8) | 7 (28)  14 (56)  4 (16) | 13 (32.5)  17 (42.5)  10 (25) | 114 (25.2)  196 (43.4)  142 (31.4) |
| Number of Co-morbidities ^b^  0  1  >=2 | 21 (31.8)  20 (30.1)  25 (37.9) | 12 (48)  8 (32)  5 (20) | 9 (22.5)  13 (32.5)  18 (45) | 147 (32.5)  144 (31.8)  161 (35.6) |
| Asymptomatic patients with family history for VTE or thrombophilia in 1^st^ grade relative (N= 504) | | | | |
|  | Low-Risk Hereditary Thrombophilia  n=211 | High-Risk Hereditary Thrombophilia  n=37 | Antiphospholipid antibody syndrome  n=0 | No Thrombophilia  n=256 |
| Number of thrombotic risk factors ^a^  0  1  >=2 | 146 (69.2)  49 (23.2)  16 (7.6) | 23 (62.2)  10 (27.1)  4 (10.8) | 0  0  0 | 161 (62.9)  75 (29.3)  20 (7.8) |
| Number of Co-morbidities ^b^  0  1  >=2 | 163 (77.3)  35 (16.6)  13 (6.2) | 33 (89.1)  4 (10.8)  0 | 0  0  0 | 196 (76.6)  40 (15.6)  20 (7.8) |
| Patients with pregnancy-related morbidity (N= 121) | | | | |
|  | Low-Risk Hereditary Thrombophilia  n=31 | High-Risk Hereditary Thrombophilia  n=18 | Antiphospholipid antibody syndrome  n=12 | No Thrombophilia  n=59 |
| Number of thrombotic risk factors ^a^  0  1  >=2 | 20 (64.5)  6 (19.4)  5 (16.1) | 10 (55.6)  3 (16.7)  5 (27.8) | 7 (58.3)  2 (16.7)  3 (25) | 42 (71.2)  13 (22)  4 (6.8) |
| Number of Co-morbidities ^b^  0  1  >=2 | 26 (84.9)  4 (12.9)  1 (3.2) | 14 (77.8)  2 (11.1)  2 (11.1) | 7 (58.4)  3 (25)  2 (16.7) | 45 (76.3)  11 (18.6)  0 |

Abbreviations: ATE, arterial thrombosis; VTE, venous thromboembolism. P value is not shown because all differences between the groups using χ2 test were insignificant (P > 0.05)

1. Risk factors include smoking, immobilization>4h, active cancer, central intravenous catheter, infection requiring bedrest with bathroom privileges only, estrogen-based treatment, pregnancy, active cancer, obesity ≥ 30 kg/m2, trauma, surgery requiring systemic anesthesia
2. Co-morbidities include diabetes mellitus, arterial hypertension, presence of any severity of liver cirrhosis, presence of chronic kidney disease, rheumatic diseases, depression, dyslipidemia, pulmonary, neurological and cardiovascular morbidity, and inflammatory bowel disease

**Supplementary Table 3.** Effect measures between clinical characteristics of patients and positivity for low- and high-risk thrombophilia in patients referred because of venous thromboembolism

|  | **Low-Risk Hereditary Thrombophilia**  **Crude OR (95% CI)** | **High-Risk Hereditary Thrombophilia**  **Crude OR (95% CI)** | **Antiphospholipid Antibody Syndrome**  **Crude OR (95% CI)** |
| --- | --- | --- | --- |
| Thrombotic risk factors | | | |
| Smoking | 1.13(0.75 – 1.51) | 0.92 (0.49 – 1.39) | 1.84 (1.21 - 2.78) |
| Immobilisation > 4 hours | 0.98 (0.70 – 1.19) | 1.37 (0.97 – 1.73) | 0.71 (0.43 - 1.17) |
| Infection | 0.91 (0.49 – 1.69) | 0.79 (0.32 – 2.43) | 1.13 (0.35 - 3.62) |
| Estrogen-based medication | 1.27 (1.01 – 1.60) | 1.33 (0.92 – 1.93) | 0.53 (0.30 - 0.93) |
| Pregnancy | 0.75 (0.42 – 1.41) | 1.49 (0.66 – 3.45) | 1.41 (0.64 - 3.07) |
| Central intravenous catheter | 0.22 (0.03 – 1.66) | 0.87 (0.11 – 6.59) | † |
| Active cancer | 0.59 (0.32 – 1.09) | 0.45 (0.15 – 1.76) | 0.53 (0.13 - 2.16) |
| Obesity (BMI > 30 kg/m2) | 0.95 (0.70 – 1.17) | 0.83 (0.53 – 1.29) | 0.86 (0.51 - 1.44) |
| Trauma | 1.41 (0.93 – 2.14) | 0.89 (0.40 – 1.94) | 0.88 (0.32 - 2.43) |
| Surgery | 0.57 (0.38 – 0.81) | 1.19 (0.69 - 1.65) | 0.51 (0.19 - 1.40) |
| Cancer medication | 0.50 (0.19 – 1.48) | 1.42 (0.43 – 4.73) | † |
| Extended varicosis | 0.79 (0.48 – 1.29) | 0.94 (0.43 – 2.07) | 1.06 (0.46 - 2.44) |
| PFO or another cardiac septal defect | 0.83 (0.22 – 2.25) | † | 2.11 (1.19 - 3.75) |
| Post Partum period | 0.58 (0.17 – 1.99) | 0.70 (0.09 – 5.23) | 1.26 (0.17 - 9.38) |
| Co-morbidities | | | |
| Diabetes mellitus | 0.42 (0.21 – 0.81) | 0.73 (0.29 – 1.82) | 0.63 (0.20 - 2.01) |
| Arterial hypertension | 0.82 (0.68 – 1.18) | 0.72 (0.44 – 1.17) | 1.04 (0.64 - 1.69) |
| Liver cirrhosis | 0.52 (0.15 – 1.76) | 1.57 (0.32 – 5.99) | 2.33 (0.54 - 9.94) |
| Chronic kidney disease | 0.53 (0.21 – 1.02) | 0.15 (0.02 – 1.07) | 1.11 (0.40 - 3.07) |
| Rheumatic disease | 0.72 (0.46 – 1.11) | 0.45 (0.18 – 1.11) | 3.30 (2.00 - 5.22) |
| Depression | 0.88 (0.52 – 1.37) | 0.75 (0.31 – 1.72) | 0.77 (0.31 - 1.92) |
| Chronic inflammatory bowel disease | 1.29 (0.58 – 2.77) | 0.71 (0.17 – 2.98) | 1.68 (0.52 - 5.46) |
| Dyslipidaemia | 0.93 (0.66 – 1.59) | 0.86 (0.45 – 1.62) | 0.95 (0.53 - 1.71) |
| Cardiovascular Disease ^a^ | 0.67 (0.39 – 1.19) | 0.46 (0.27 – 1.38) | 1.55 (0.77 - 3.11) |
| Pulmonal Disease ^b^ | 1.03 (0.72 – 1.46) | 0.97 (0.54 – 1.76) | 1.21 (0.64 - 2.28) |
| Neurological Disorders ^c^ | 1.32 (0.78 – 2.26) | 0.18 (0.04 – 1.26) | 1.94 (0.88 - 4.26) |

Abbreviations: ATE, arterial thrombosis; CI, confidence interval; OR, odds ratio; VTE, venous thromboembolism; ATE, arterial thromboembolism; PFO, patent foramen ovale.

ORs were calculated using multivariate regression model in hereditary thrombophilia adjusting for age < 50 years old and positive family history for VTE in 1^st^ grade relative and univariate regression in antiphospholipid antibody syndrome. Low-risk thrombophilia is defined by the presence of heterozygous factor V Leiden or heterozygous prothrombin 20210G>A mutation; high-risk hereditary thrombophilia comprises homozygous factor V Leiden, homozygous prothrombin 20210G>A mutation, PS, PC, and AT deficiencies or combined thrombophilia. †- Number of events are too small to calculate the ORs

1. Cardiomyopathy, aortopathy
2. Asthma, interstitial lung disease, obstructive lung disease, obstructive sleep apnea
3. Epilepsy, parkinson disease, multiple sclerosis

**Supplementary Table 4.** Effect measures between clinical characteristics of patients and positivity for low- and high-risk thrombophilia in patients with otherwise unexplained arterial thrombosis, pregnancy-related morbidity and asymptomatic family members with positive family history of VTE or thrombophilia in 1^st^ grade relative

| Patients with otherwise unexplained arterial thrombosis (N=583) | | |
| --- | --- | --- |
| **Clinical and Laboratory Characteristics** | **Low-risk hereditary thrombophilia**  **Crude OR (95% CI)** | **High-risk hereditary thrombophilia**  **Crude OR (95% CI)** |
| Age <50 years ^a^ | 0.87 (0.50 - 1.49) | 0.84 (0.47 - 1.91) |
| Females ^b^ | 0.73 (0.42 - 1.24) | 0.96 (0.41 - 2.15) |
| Smoking | 1.15 (0.76 - 1.95) | 0.43 (0.26 - 1.29) |
| Immobilisation > 4 hours | 1.28 (0.42 - 5.49) | 1.28 (0.19 - 9.19) |
| Infection requiring immobilisation | 0.98 (0.11 - 7.54) | † |
| Estrogen-based medication | 0.97 (0.43 - 2.47) | 1.55 (0.51 - 4.69) |
| Pregnancy | † | † |
| Central intravenous catheter | † | † |
| Active cancer | 0.53 (0.17 - 4.10) | 1.48 (0.19 - 11.63) |
| Obesity (BMI > 30 kg/m2) | 1.01 (0.68 - 2.14) | 0.51 (0.12 - 2.21) |
| Trauma | † | 7.95 (0.79 - 9.71) |
| Surgery requiring general anaesthesia | † | † |
| Cancer medication | † | † |
| Extended varicose veins | 0.49 (0.06 - 3.87) | 2.83 (0.60 - 13.33) |
| PFO or another cardiac septal defect | 0.58 (0.37 - 1.43) | 0.64 (0.25 - 1.64) |
| Post partum period | † | † |
| Positive family history for VTE in 1st grade relative | 1.57 (0.85 - 2.99) | 0.85 (0.28 - 2.66) |
| Diabetes mellitus | 0.73 (0.22 - 2.44) | 1.31 (0.30 - 5.52) |
| Arterial Hypertension | 1.07 (0.61 - 1.95) | 0.64 (0.24 - 1.77) |
| Liver cirrhosis | † | † |
| Chronic kidney disease | † | † |
| Rheumatic disease | 0.78 (0.22 - 2.57) | † |
| Depression | 0.95 (0.35 - 2.46) | 0.94 (0.21 - 4.38) |
| Chronic inflammatory bowel disease | † | † |
| Dyslipidaemia | 1.14 (0.66 - 1.96) | 0.47 (0.17 - 1.28) |
| Cardiovascular Disease ^c^ | 0.74 (0.28 - 1.94) | 1.22 (0.35 - 4.21) |
| Pulmonal Disease ^d^ | 1.72 (0.86 - 3.71) | 1.37 (0.39 - 4.66) |
| Neurological Disorders ^e^ | 0.40 (0.05 - 3.04) | † |
| D-Dimer, ug/L ≥ 500 | 0.77 (0.35 - 1.53) | 1.71 (0.67 - 4.36) |
| TAT-complex, mcg/L > 4.1 | 1.65 (0.75 - 3.59) | 2.05 (0.65 - 6.43) |
| Factor VIII, % ≥ 164 | 0.49 (0.15 - 1.34) | 1.23 (0.72 - 3.61) |
| Fibrinogen, g/L ≥ 3.75 | 0.47 (0.13 - 1.16) | 0.53 (0.12 - 2.26) |
| Homocysteine, μmol/L ≥ 15 | 1.01 (0.51 - 1.87) | 1.61 (0.58 - 4.47) |
| vWF, Ag % ≥ 136 | 0.82 (0.41 - 1.57) | 1.19 (0.31 - 4.64) |
| Asymptomatic patients with family history for VTE or thrombophilia in 1^st^ grade relative (N= 504) | | |
| **Clinical and Laboratory Characteristics** | **Low-risk hereditary thrombophilia**  **Crude OR (95% CI)** | **High-risk hereditary thrombophilia**  **Crude OR (95% CI)** |
| Age <50 years ^a^ | 1.21 (0.67 - 2.20) | 2.39 (0.56 - 10.28) |
| Females ^b^ | 1.15 (0.72 - 1.82) | 1.64 (0.66 - 4.22) |
| Smoking | 0.61 (0.34 - 1.24) | 2.80 (1.24 - 6.30) |
| Immobilisation > 4 hours | 0.57 (0.04 - 3.52) | † |
| Infection requiring immobilisation | † | † |
| Estrogen-based medication | 0.17 (0.08 - 0.39) | 1.94 (0.79 - 4.54) |
| Pregnancy | 1.61 (0.84 - 3.08) | 0.94 (0.27 - 3.25) |
| Central intravenous catheter | † | † |
| Active cancer | 5.26 (0.47 - 47.47) | † |
| Obesity (BMI > 30 kg/m2) | 1.15 (0.52 - 2.69) | † |
| Trauma | † | † |
| Surgery requiring general anaesthesia | 0.73 (0.07 - 8.06) | † |
| Cancer medication | † | † |
| Extended varicose veins | 0.56 (0.18 - 1.95) | 2.23 (0.45 - 10.46) |
| PFO or another cardiac septal defect | † | † |
| Post partum period | † | † |
| Positive family history for VTE in 1st grade relative | 1.15 (0.73 - 1.59) | 1.28 (0.63 - 2.35) |
| Diabetes mellitus | 2.30 (0.20 - 26.82) | † |
| Arterial Hypertension | 0.64 (0.24 - 1.44) | † |
| Liver cirrhosis | † | † |
| Chronic kidney disease | † | † |
| Rheumatic disease | 1.14 (0.42 - 3.10) | 0.82 (0.10 - 6.33) |
| Depression | 0.86 (0.29 - 2.46) | † |
| Chronic inflammatory bowel disease | 1.46 (0.23 - 10.45) | † |
| Dyslipidaemia | 0.30 (0.08 - 1.11) | 0.75 (0.09 - 6.42) |
| Cardiovascular Disease ^c^ | 0.35 (0.04 - 3.25) | † |
| Pulmonal Disease ^d^ | 0.52 (0.19 - 1.41) | 0.52 (0.07 - 4.20) |
| Neurological Disorders ^e^ | 0.76 (0.22 - 2.67) | † |
| D-Dimer, ug/L ≥ 500 | 1.71 (0.88 - 3.52) | 1.42 (0.38 - 5.29) |
| TAT-complex, mcg/L > 4.1 | 2.16 (1.03 - 4.52) | 1.83 (0.49 - 7.27) |
| Factor VIII, % ≥ 164 | 1.27 (0.50 - 3.10) | 1.46 (0.29 - 7.26) |
| Fibrinogen, g/L ≥ 3.75 | 1.45 (0.78 - 2.70) | 0.42 (0.12 - 2.44) |
| Homocysteine, μmol/L ≥ 15 | 3.48 (1.26 - 10.14) | † |
| Patients with pregnancy-related morbidity (N= 120) | | |
| **Clinical and Laboratory Characteristics** | **Low-risk hereditary thrombophilia**  **Crude OR (95% CI)** | **High-risk hereditary thrombophilia**  **Crude OR (95% CI)** |
| Age <50 years ^a^ | † | † |
| Females ^b^ | † |  |
| Smoking | 0.96 (0.18 - 5.36) | † |
| Immobilisation > 4 hours | † | † |
| Infection requiring immobilisation | † | † |
| Estrogen-based medication | † | 5.76 (0.34 - 96.54) |
| Pregnancy | 1.30 (0.45 - 3.77) | 1.49 (0.43 - 5.09) |
| Central intravenous catheter | † | † |
| Active cancer | † | † |
| Obesity (BMI > 30 kg/m2) | † | 0.49 (0.05 - 4.39) |
| Trauma | † | † |
| Surgery requiring general anaesthesia | † | † |
| Cancer medication | † | † |
| Extended varicose veins | 2.00 (0.32 - 12.69) | 0.86 (0.09 - 8.84) |
| PFO or another cardiac septal defect | † | † |
| Post partum period | † | † |
| Positive family history for VTE in 1st grade relative | 0.97 (0.35 - 2.52) | 2.71 (0.97 - 7.61) |
| Diabetes mellitus | † | † |
| Arterial Hypertension | † | 1.88 (0.18 - 19.16) |
| Liver cirrhosis | † | † |
| Chronic kidney disease | † | † |
| Rheumatic disease | 0.97 (0.16 - 9.65) | † |
| Depression | † | † |
| Chronic inflammatory bowel disease | † | † |
| Dyslipidaemia | † | † |
| Cardiovascular Disease ^c^ | † | † |
| Pulmonal Disease ^d^ | † | † |
| Neurological Disorders ^e^ | † | † |
| D-Dimer, ug/L ≥ 500 | 1.93 (0.52 - 7.21) | 0.60 (0.07 - 5.19) |
| TAT-complex, mcg/L > 4.1 | 1.24 (0.29 - 5.24) | † |
| Factor VIII, % ≥ 164 | 2.25 (0.49 - 10.62) | † |
| Fibrinogen, g/L ≥ 3.75 | 0.34 (0.04 - 2.84) | 0.57 (0.07 - 4.81) |
| Homocysteine, μmol/L ≥ 15 | † | † |
| vWF, Ag % ≥ 136 | † | † |

Abbreviations: ATE, arterial thrombosis; CI, confidence interval; OR, odds ratio; VTE, venous thromboembolism; ATE, arterial thromboembolism; PFO, patent foramen ovale; vWF Ag, von Willebrand factor antigen.

ORs were calculated using multivariate regression model in hereditary thrombophilia adjusting for age < 50 years old and positive family history for VTE in 1^st^ grade relative. Too little events were present to perform a univariate regression in antiphospholipid antibody syndrome. Low-risk thrombophilia is defined by the presence of heterozygous factor V Leiden or heterozygous prothrombin 20210G>A mutation; high-risk hereditary thrombophilia comprises homozygous factor V Leiden, homozygous prothrombin 20210G>A mutation, PS, PC, and AT deficiencies or combined thrombophilia.

1. Age at consultation in asymptomatic patients, age at time of thrombotic event or pregnancy-related morbidity in other patients’ groups
2. In reference to males
3. Cardiomyopathy, aortopathy
4. Asthma, interstitial lung disease, obstructive lung disease, obstructive sleep apnea
5. Epilepsy, parkinson disease, multiple sclerosis
